# Postpartum doula care and symptoms of depression and anxiety among BIPOC and Latina birthing parents: a longitudinal pilot intervention study

**DOI:** 10.3389/fgwh.2026.1869956

**Published:** 2026-09-01

**Authors:** Jessica Hanlin, Laurel M. Hicks, Gabriela Alvarado, Alison Batwin, Michele Holland, Elizabeth Greenwell

**Affiliations:** 1 Sanctuary CARES, Lafayette, CO, United States; 2 Sanctuary Doulas & Family Care, Lafayette, CO, United States; 3 Renée Crown Wellness Institute, University of Colorado-Boulder, Boulder, CO, United States; 4 Rural Health Institute, University of Wyoming, Laramie, WY, United States; 5 Sanctuary Healing Arts, Longmont, CO, United States; 6 Colorado School of Public Health, University of Colorado Anschutz Medical Campus, Aurora, CO, United States

**Keywords:** community-based, doula care, health equity, maternal mental health, perinatal mental health, postpartum, postpartum doula

## Abstract

**Background:**

Perinatal mental health conditions are leading complications in pregnancy and postpartum in the United States, and Black, Indigenous, People of Color (BIPOC) and Latina birthing parents experience disproportionate postpartum mental health burdens and care inequities. Set within a high-income U.S. state, this study examines a population experiencing socioeconomic and structural barriers that may contribute to postpartum mental health vulnerability. Most participants were Medicaid beneficiaries facing barriers, including food insecurity, limited social support, and limited healthcare access. We explored the impact of postpartum doula care on symptoms of depression and anxiety in this cohort of BIPOC and Latina birthing parents as well as participant satisfaction, acceptability, and feasibility of the intervention.

**Methods:**

In this single-arm longitudinal pilot (*N* = 33), we investigated the effects of postpartum doula care on mental health markers among a cohort of birthing parents reporting elevated symptoms of depression or anxiety. This non-medical intervention consisted of in-home, postpartum doula care visits (∼24 h total, typically six visits of four hours each) within the first year postpartum. Data were collected at three timepoints (T1-baseline, T2-mid-study, T3-end point).

**Results:**

Paired t-tests among participants completing all study assessments (*n* = 24) demonstrated significant reductions in both depression and anxiety levels from T1 to T3. The intervention was successfully delivered, with participants completing the study receiving nearly the full intended dose of postpartum doula care. Participants reported high satisfaction, including perceived benefits to their postpartum experience, mood, and anxiety, and strong acceptability of postpartum doula care for future use.

**Conclusion:**

Although larger, controlled studies are needed, these findings suggest that postpartum doula care is a promising strategy that warrants further evaluation for effectiveness, scalability and advancing perinatal mental health equity.

## Introduction

Perinatal mental health (PMH) conditions are the most common complication of pregnancy and are a leading contributor to maternal morbidity and mortality in the United States (U.S.), despite the country's status as a high-income and comparatively well-resourced nation. This burden of perinatal mental health conditions is not distributed evenly across populations, with Black and Latina birthing parents experiencing higher risks of postpartum depressive symptoms and persistent inequities in access to postpartum mental health care compared with their white counterparts (1). With over 42% of births in the U.S. covered by Medicaid (2), the country's public insurance system for low-income residents, socioeconomic and structural disadvantages are widespread (3). Socioeconomic disadvantage is strongly associated with increased rates of PMH conditions, and women from low-income and ethnic minority groups are at increased risk of depression and anxiety (4) with prevalence rates of depression reaching as high as 38% (5). Barriers accessing medical care, food insecurity, limited social support, and residence in lower-socioeconomic status neighborhoods intersect with structural racism and historical medical distrust. Together, these factors contribute significantly to elevated perinatal mental health vulnerability within this population.

Research indicates that while the national prevalence of PMH conditions is approximately 15%–20%, rates among Black women have been reported as high as 56% in some urban settings (6), with broader estimates across multiple studies ranging from 35 to 67% among racial and ethnic minorities across the U.S (7–9). In Colorado specifically, a study of the vulnerable, primarily Hispanic population served at a federally qualified health center found the prevalence of PMH conditions was nearly double the nationally reported rate (10). Furthermore, the Colorado Maternal Mortality Review Committee identified suicide and mental health concerns as the primary cause of pregnancy-related deaths in the state (11, 12). While the clinical community has increasingly recognized these gaps, traditional interventions and medical care have yet to address the holistic needs of the birthing parent in their home environment, which are deeply intertwined with postpartum mental health recovery.

One promising but under-researched intervention is the utilization of postpartum doulas. Differing from birth doulas, who provide education and support during pregnancy, labor and delivery, postpartum doulas provide home-based, non-medical support to facilitate the physical and emotional transition of the entire family after delivery. Their comprehensive scope of practice includes assisting with birthing parent recovery, newborn care, and household organization, while fostering evidence-based learning and providing referrals as necessary (13). In contrast to clinic-based models of postpartum follow-up, postpartum doula care is delivered where recovery and parenting are actually taking place: in the home, amid the social, emotional, and material realities of the postpartum period. The positioning may be particularly important for families facing barriers to transportation, fragmented care systems, limited social support, or competing work and caregiver demands.

To date, existing literature has focused heavily on birth doulas and their impact on obstetric outcomes (such as cesarean birth rates and labor duration), with substantial qualitative research existing on the positive impact of non-medical postpartum social and practical support. This includes non-medical professionals, community, and family. This kind of postpartum social support improves outcomes in mothers’ mental health, including less depression and anxiety (14), more success in breastfeeding (15), improved confidence as a new parent (16), and a lower incidence of abuse than those who do not receive such support (17). Many of these reflect domains that are often under-addressed in biomedical postpartum care, including practical household relief, emotional presence, rest facilitation, and navigation of daily caregiving demands. A 2024 scoping review highlighted a critical gap in the field, calling for more quantitative, longitudinal evidence to substantiate the impact of postpartum doula care (18).

In response to this evidence gap, we used a single-arm, longitudinal pilot intervention model to examine the impact of postpartum doula care on symptoms of anxiety and depression in a cohort of BIPOC and Latina birthing parents in Colorado. We specifically chose this geographic area due to the substantive postpartum doula workforce in and around the Boulder/Denver metro area as well as the adequate population diversity (approximately 35% identifying as BIPOC and/or Latina) to reach our sample size. We also aimed to evaluate the intervention's satisfaction, acceptability, and feasibility in an effort to support future studies focused on closing the gap. We hypothesized that depression and anxiety symptoms would improve from baseline to the study endpoint and that the intervention would generally be accepted by the participants involved. By focusing on a population disproportionately burdened by PMH conditions and by testing a home-based, non-medical model of support, this study also sought to inform equity-oriented perinatal health strategies that extend beyond traditional medical settings and into the postpartum environments where many unmet needs remain concentrated.

## Materials and methods

### Design and setting

We employed a longitudinal pilot intervention study (*N* = 33) with BIPOC and Latina birthing parents who self-reported experiencing depression or anxiety symptoms during pregnancy or after childbirth to explore the impact of postpartum doula care on these two perinatal mental health markers. The primary aims were to assess preliminary changes in mental health symptoms over time and to evaluate the acceptability, satisfaction, and feasibility of the intervention.

**Figure 1:**
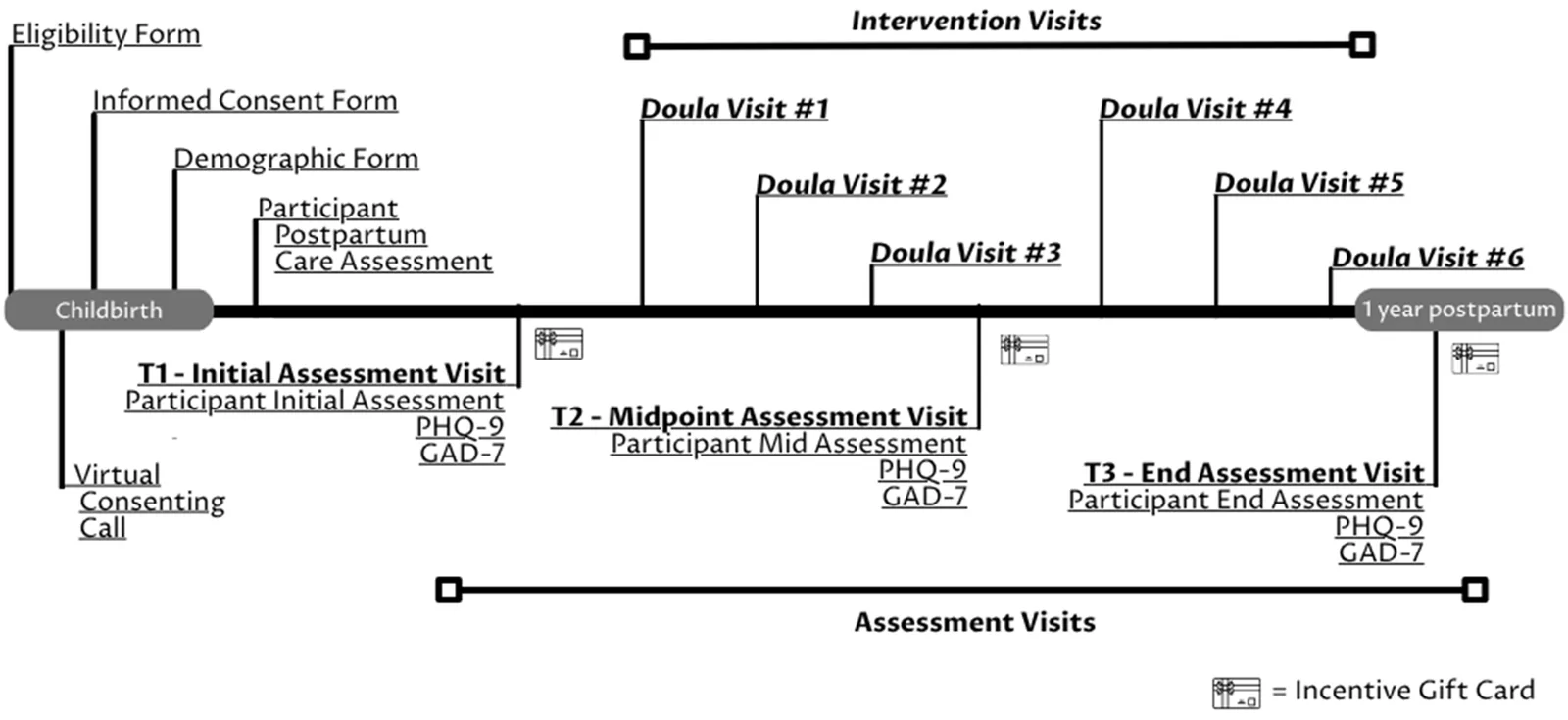
Study flow and longitudinal assessment framework.

The study's intervention consisted of a series of postpartum doula care visits in the birthing parent's home (24 h of care provided total, generally offered in six, 4-hour-long visits) occurring within the first year postpartum. Assessment visits were completed at three time points (see Figure 1): T1 (baseline, prior to first intervention visit), T2 (mid-study) and T3 (end point, after final intervention visit).

All assessment and intervention visits occurred between January 2024 and August 2025 in each participant’s home in the Denver/Boulder metropolitan area of Colorado, U.S. All study protocols and activities received ethical oversight and approval from Advarra, a private institutional review board (IRB).

### Participants and recruitment

Our recruitment efforts included reaching prospective study participants by advertising via physical flyers in locally established prenatal and postpartum health care provider offices, agencies, and hospitals, as well as online and through social media platforms. All recruitment materials were presented in both English and Spanish in order to actively recruit both English and Spanish speakers. Physical flyers included a QR code leading to the study's website that outlined eligibility criteria, study procedures, and incentives. Interested participants completed an eligibility form that ensured they met inclusion criteria. Those who were eligible then completed a virtual or phone consenting call with the study's research coordinator in which the study's informed consent form was reviewed, including study procedures, potential study benefits, risks, and study incentives.

**Inclusion criteria were:** (1) self-identification as Black, Indigenous, Asian, Native Hawaiian/Pacific Islander race and/or Hispanic ethnicity, (2) currently pregnant and/or will be no more than one year postpartum prior to the end of data collection, as well as one of the following: (A) self-report of an assessment and/or diagnosis of depression or anxiety made by their medical provider in this pregnancy or first year postpartum, or (B) a score of five or more on the Patient Health Questionnaire (PHQ-9) assessment included in the Eligibility Form.

**Exclusion criteria were:** (1) already receiving or planning to receive postpartum doula support during the first year postpartum, (2) receiving psychiatric care in an in-patient or partial hospitalization setting, (3) self-reporting domestic violence at home, as this presents a safety issue for research and doula intervention personnel entering the home. These exclusion criteria were reviewed during eligibility determination only and do not include any outpatient mental health therapy or medication support. Those who met the exclusion criteria were referred to appropriate local, county and community support programs.

Participants then returned their signed consent forms after the virtual consenting call. The study's participation incentive consisted of a series of gift cards distributed after each assessment visit, up to a total of $100. Spanish translations of all study materials including recruitment flyers, eligibility form, informed consent form, other forms, and study scripts were available to meet the needs of our Spanish speaking participant population.

### Intervention components

The intervention utilized a standardized yet flexible delivery model, ensuring that while five categorical pillars of postpartum doula care (defined below) remained constant, the specific “dosage” of each component was tailored to the participant's unique socioeconomic stressors and household needs throughout the first year postpartum. We categorized support into: (1) *Recovery and Emotional Well-being of the Birthing Parent* - focusing on physical healing and offering emotional support; (2) *Sleep and Rest Optimization* - facilitating restorative rest to mitigate physiological and psychological triggers; (3) *Newborn Care and Feeding Education* - aimed at increasing parental self-efficacy; (4) *Household Support* - offered through organizational and nutritional support; and (5) *Resource Navigation and Advocacy* - empowering participants to navigate fragmented medical and social service landscapes (19). By addressing both the immediate physical, emotional, and logistical demands of the first year postpartum and the broader structural barriers facing BIPOC and Latina families, the intervention provided a comprehensive framework designed to reduce the cumulative stress associated with perinatal mental health vulnerability. This structure was intended to preserve intervention fidelity at the level of core domains while allowing responsiveness to participant-defined needs within each household context. Upon completion of each postpartum doula visit, the doula recorded what percentage of the visit was spent facilitating each of the first four categories of support and then indicated if they assisted with resource navigation during that visit or not.

The postpartum doulas were professional, non-medical providers trained to deliver emotional, informational, and practical support rather than psychotherapy or clinical treatment. The postpartum doulas met professional training standards, were vetted through background checks, had completed infant CPR certification, and had access to ongoing peer support, leadership oversight and supervision of care provided through Sanctuary Doulas & Family Care. When needed, postpartum doulas reinforced referral pathways to medical or mental health services, but the intervention itself was not designed as a clinical mental health treatment. This distinction is important in interpreting the findings, as the study evaluates whether non-medical, home-based support may contribute to improved mental health symptoms in the postpartum period. The postpartum doulas were blinded to participants’ PHQ-9 and GAD-7 scores throughout the study to ensure that care was delivered based on the doula's assessment of needs in the visit and not influenced by participants’ reported levels of depression or anxiety.

### Measures

Pre-visit assessments were sent electronically to study participants and included the study's demographic form and participant postpartum care assessment. In these self-report questionnaires, we assessed already established and/or existing postpartum support, socioeconomic indicators of stress, risk factors for depression and anxiety, other personal and family history of mental health, and demographic information.

Mental health outcomes were measured using the heavily validated screening tools Patient Health Questionnaire (PHQ-9) and the Generalized Anxiety Disorder-7 (GAD-7) to gather quantitative measurements of maternal levels of depression and anxiety. The PHQ-9 is a self-administered, nine-item questionnaire that screens for depression symptoms and severity, with scoring from 0 to 27 (20). The questionnaire asks “Based on your experience over the last 2 weeks, how often have you been bothered by any of the following problems?” and participants choose a number from 0 to 3 (0 represents “not at all” to 3 “nearly every day”) (21). Although the PHQ-9 was originally developed and normed in primary care settings, subsequent research has supported its validity as a perinatal depression screener, with a systematic review and meta-analysis demonstrating high sensitivity and specificity of 0.81 across perinatal populations (20). We acknowledge, however, that self-report measures may underestimate symptom burden in low-income Black and Latina populations due to culturally influenced disclosure patterns and medical mistrust, and this should be considered when interpreting findings (22).

The GAD-7 questionnaire is a self-administered, seven-item questionnaire that identifies probable generalized anxiety disorder along with measuring severity (23). The GAD-7 uses the same question and scoring model as the PHQ-9, but scoring ranges from 0 to 21. The study participants also completed the applicable participant initial, mid- and post- intervention assessments during respective study visits. These questionnaires also utilized open-ended questions to assess acceptability and feasibility. Although the EPDS-3A is a valid measure to screen for anxiety (24), the GAD-7 was chosen as it aligns with all of the symptom criteria in the DSM-5 and has a higher detection of perinatal anxiety (23).

**Acceptability assessments asked**: i) what study participants know about postpartum doula care prior to receiving the intervention, ii) how they feel about receiving doula care in the future, and iii) potential barriers and stressors that might impact their experience in receiving the intervention.

**Feasibility assessments by asked**: i) the doula service provider’s history and infrastructure, ii) referral timing related to when the participant gave birth, and iii) challenges with participant communication and follow-up.

### Analysis

Data analysis was performed using IBM SPSS version 29.0 (25). Prior to conducting the analyses, data were examined statistically and graphically to verify quality of data and identify potential univariate outliers. There was no evidence of incorrect values or univariate outliers. Missing data were due to individual measures that were not completed by some study participants. Baseline demographics and descriptive characteristics were calculated for all outcome data. Paired t-tests with listwise deletion were administered to compare means between time points for PHQ-9 and GAD-7 assessment data. Open-ended questions were reviewed descriptively to capture participant-reported experiences with the intervention. Responses were brief and were examined to identify commonly noted words and themes as a preliminary, exploratory exercise. This was not intended as a formal qualitative analysis but rather as a supplementary observation to contextualize quantitative findings. Analyses were intended to estimate preliminary patterns of change and generate effect size information rather than establish causal inference. We therefore interpret findings as hypothesis-generating and supportive of future studies.

## Results

Thirty-three BIPOC and Latina birthing parents met eligibility criteria, were enrolled; twenty-four completed all study visits. The nine people who did not complete the study were lost to follow up and didn't give a reason for not completing the assessments. Participants received a mean of 23.67 h of postpartum doula care (min 18, max 24) with the intervention dose designed to be 24 h of care. The average number of days between T1 and T3 was 81.7 days, with the average between T1 and T2 being 39 days, and between T2 and T3 being 42.7 days. Findings are organized to first characterize the study sample, then describe changes in depression and anxiety outcomes across the three timepoints, and finally summarize engagement with and acceptability of the postpartum doula intervention.

### Participant characteristics

Thirty-three participants were enrolled in the study, meeting full eligibility criteria (PHQ-9 score ≥ 5, or a self-reported medical diagnosis of depression or anxiety) and included in the primary analysis (See Table 1). The sample was racially and ethnically diverse, reflecting the equity focus of the study. Nearly half of participants (48.5%) identified as Latina/Hispanic, and 27.3% identified as Black. Smaller proportions identified as Asian (12.1%), Indigenous/Native American (3.0%), or other/multiracial (9.0%). The majority of participants (57.6%) identified as Hispanic in ethnicity, with 42.4% identifying as Non-Hispanic. All participants (100%) identified as female.

**Table 1:** Participant demographic and health characteristics.

	Full sample (*N* = 33)	Paired *t*-test sample only (*n* = 24)
Characteristic	*n* (%)	*n* (%)
Age		
Under 20 years	1 (3.0)	1 (4.2)
20–24 years	8 (24.2)	5 (20.8)
25–29 years	9 (27.3)	8 (33.3)
30–34 years	7 (21.2)	5 (20.8)
35–39 years	3 (9.1)	2 (8.3)
40–49 years	2 (6.1)	2 (8.3)
Missing	3 (9.1)	1 (4.2)
Race/Ethnicity		
Latina/Hispanic	16 (48.5)	11 (45.8)
Black	9 (27.3)	7 (29.2)
Asian	4 (12.1)	4 (12.1)
Indigenous/Native American	1 (3.0)	0 (0.0)
Other/Multiracial	3 (9.0)	2 (8.3)
Ethnicity		
Hispanic	19 (57.6)	13 (54.2)
Non-Hispanic	14 (42.4)	11 (45.8)
Education		
Did not complete high school	1 (3.0)	0 (0.0)
High school graduate	6 (18.2)	5 (20.8)
Some college/university	12 (36.4)	9 (37.5)
College/university graduate	6 (18.2)	5 (20.8)
Graduate level	5 (15.2)	4 (16.7)
Missing	3 (9.1)	1 (4.2)
Insurance		
Medicaid	21 (63.6)	16 (66.7)
Commercial insurance	6 (18.2)	5 (20.8)
No insurance	2 (6.1)	1 (4.2)
Don't know	1 (3.0)	1 (4.2)
Missing	3 (9.1)	1 (4.2)
Employment^a^		
Unemployed	13 (39.4)	9 (37.5)
Employed full-time	7 (21.2)	5 (20.8)
Employed part-time	6 (18.2)	5 (20.8)
Student	5 (15.1)	4 (16.7)
Missing	3 (9.1)	1 (4.2)
Mental Health History		
History of depression	21 (63.6)	17 (70.8)
Current depression	14 (42.4)	11 (45.8)
History of anxiety	24 (72.7)	18 (75.0)
Current anxiety	22 (66.7)	16 (66.7)
Family history of mental illness	15 (45.4)	12 (50.0)
Mental Health Reported for Eligibility		
PHQ-9 > 5	30 (90.1)	
Depression or Anxiety diagnosis	22 (66.7)	
Both endorsed	19 (57.6)	

a Employment status assessed using a checkbox format; participants could select more than one category. One reported they were both a student and employed.

Participant ages were nearly three quarters between 20 and 34 years of age. Of these, 24.2% were 20–24 years, 27.3% were 25–29 years, and 21.2% were 30–34 years. About half of the participants (45.5%) were pregnant for the first time, and a quarter (27.3%) had experienced two pregnancies. A majority (57.6%) had one child under 21 living in the home. All participants were within one year postpartum (*M* = 95.7 days, *SD* = 94.7, range 2–327 days).

Participants reflected socioeconomic indicators associated with increased postpartum mental health vulnerability. The majority (63.6%) were covered by Medicaid (the U.S. program that provides health coverage to low-income individuals and families), 6.1% were not insured, and only 18.2% had private/commercial insurance. Unemployment was prevalent, with 39.4% reporting being unemployed at the time of enrollment. The highest level of education varied: 3.0% did not complete high school, 18.2% had graduated high school, 36.4% had some college coursework, 18.2% held a college degree, and 15.2% had attained graduate-level education. Approximately one-third (30.2%) of participants reported difficulty accessing medical care, with transportation, insurance limitations, childcare needs, and lifestyle changes following birth cited as the primary barriers.

Participants in the study had a substantial mental health burden at baseline. A self-reported history of depression was reported by 63.6%, and 42.4% reported a current diagnosis of depression; 72.7% reported a history of anxiety, and 66.7% reported current anxiety. Additionally, 45.4% had a family history of mental illness. Of note, 18% indicated they received psychotherapy and 30% were taking psychotropic medication–concurrently with the intervention.

### Depression and anxiety outcomes

Among the analytic sample (*N* = 33), mean PHQ-9 scores decreased from 9.55 (*SD* = 4.84) at baseline (T1) to 5.70 (*SD* = 3.57) at mid-study (T2) and 3.44 (*SD* = 3.57) at endpoint (T3).

Mean GAD-7 scores followed a similar trajectory amongst the same sample (*N* = 33): scores declined from 8.39 (*SD* = 4.24) at baseline (T1) to 5.15 (*SD* = 3.83) at mid-study (T2) and 3.56 (*SD* = 3.68) at endpoint (T3).

The magnitude of change also appears clinically meaningful. Using established PHQ-9 cut-point thresholds, mean scores shifted from the “mild” to “moderate” range at baseline to below the clinical threshold for mild depression at endpoint. Similarly, mean GAD-7 scores moved from the “mild anxiety” range at baseline to below threshold for minimal anxiety at endpoint.

**Figure 2:**
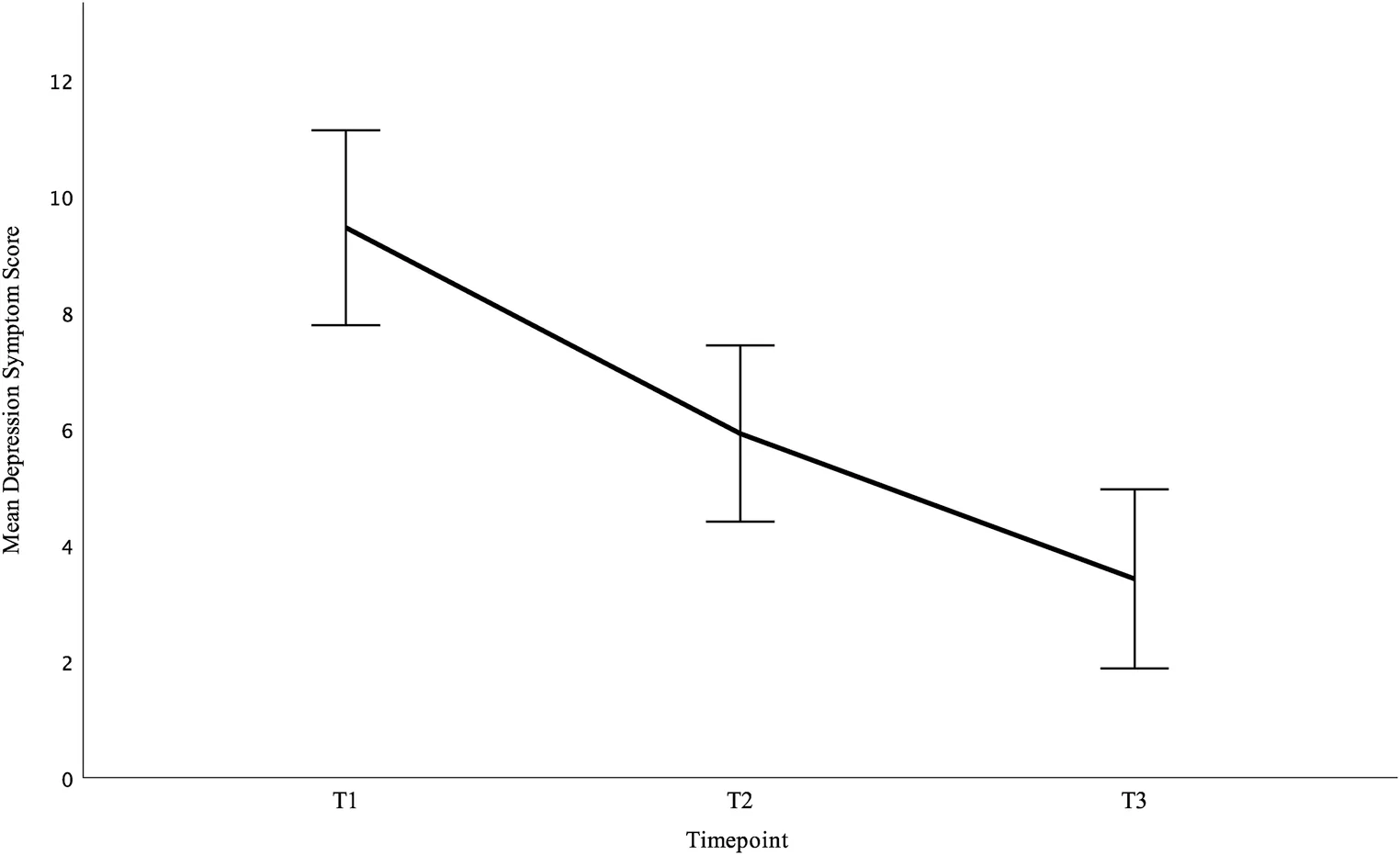
Total mean depression symptom score from pre to post postpartum intervention (*n*=24). This figure represents the mean depression symptoms (PHQ-9) score changes over time. T1 is before the postpartum doula intervention, T2 is after three postpartum doula sessions, T3 is after six postpartum doula sessions. Error bars show 95% confidence interval.

**Figure 3:**
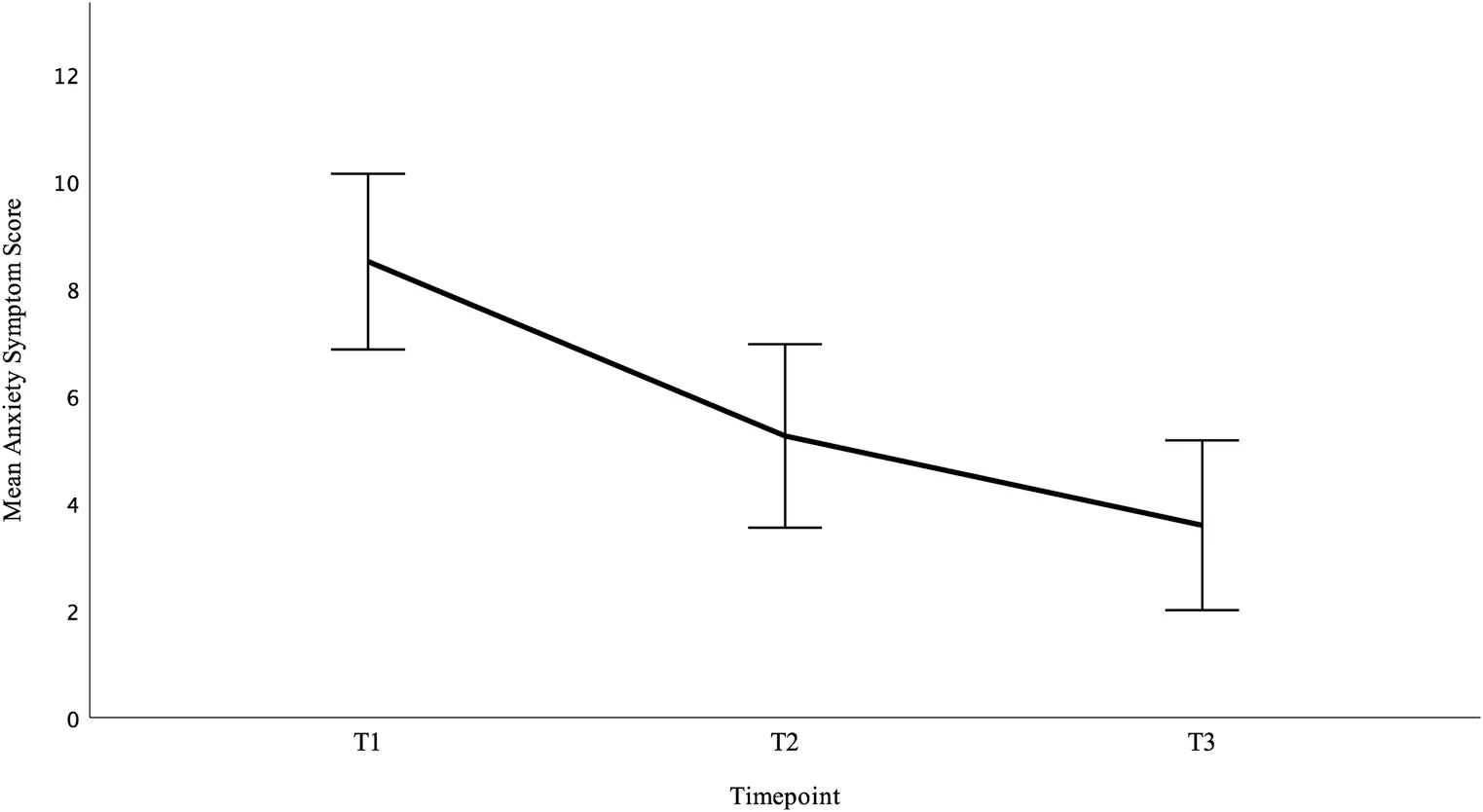
Total mean anxiety symptom score from pre to post postpartum doula intervention. This figure represents the mean anxiety (GAD-7) total score changes over time. T1 is before the postpartum doula intervention, T2 is after three postpartum doula sessions, T3 is after six postpartum doula sessions. Error bars show 95% confidence interval.

A paired-samples t-test (see Table 2) was conducted to determine whether there was a statistically significant mean difference in depression and anxiety symptoms between pre (T1) and post (T3) intervention among participants that completed all study visits (see Figures 2, 3). These three pairwise comparisons across timepoints were not corrected in the analysis, however, all *p*-values were well below conventional thresholds and remained significant under Bonferroni correction (adjusted *α* = .017) (26), reducing concern that significant findings reflect Type I error.

**Table 2:** Paired *t*-test^a^; PHQ-9 and GAD-7 scores across T1 (baseline), T2 (mid-study) and T3 (endpoint).

Comparison	Mean change	95% CI	*t*	*p*	Cohen's *d*
PHQ-9: T1 → T2	−3.54	[1.90, 5.19]	4.45	<.001	0.91
PHQ-9: T2 → T3	−2.50	[1.02, 3.98]	3.50	<.001	0.71
**PHQ-9: T1 → T3**	**−6**.**03**	**[3.94, 8.15]**	**5**.**94**	**<**.**001**	**1**.**21**
GAD-7: T1 → T2	−3.25	[1.49, 5.01]	3.82	<.001	0.78
GAD-7: T2 → T3	−1.67	[0.41, 2.92]	2.75	.006	0.56
**GAD-7: T1 → T3**	**−4**.**92**	**[3.19, 6.64]**	**5**.**90**	**<**.**001**	**1**.**20**

a (*n* = 24, *df* = 23), used listwise deletion.

Bolded rows and values indicate the overall change from T1 (baseline) to T3 (endpoint).

### Satisfaction, acceptability and feasibility

Participant satisfaction with the postpartum doula intervention was high across both mid- and endpoint assessments (See Table 3). At T2, 92.6% of participants either agreed or strongly agreed that postpartum doula care had positively impacted their experience as a postpartum parent. Two participants (7.4%) strongly disagreed. At T3, these proportions slightly increased, with 96.0% agreeing or strongly agreeing. Regarding mood specifically, all participants agreed or strongly agreed at T2 and all but one (96.0%) at T3 that doula care had improved their overall mood. With respect to anxiety, 85.1% agreed or strongly agreed at T2 and 92.0% at T3 that doula care had decreased their overall anxiety.

**Table 3:** Intervention satisfaction and acceptability at T2 and T3.

Item	*N*	Mean	Std. Dev.
Doula care positively impacted postpartum experience			
T2 timepoint	27	3.56	1.086
T3 timepoint	25	3.76	0.831
Doula care improved overall mood			
T2 timepoint	27	3.78	0.424
T3 timepoint	24	3.83	0.381
Doula care decreased overall anxiety			
T2 timepoint	27	3.22	0.934
T3 timepoint	25	3.36	1.114

When asked which category of doula support had been most impactful, household support (meal preparation, laundry, light householding, errands) was most frequently selected at T2 (33.3%), followed by parent care (emotional well-being, physical recovery, nutrition, self-care, and exercise) (18.2%) and sleep and rest support (15.2%). At T3, household support was also most frequently selected (27.3%), followed by newborn care, feeding, and education (24.2%). Referrals to medical care and community resources were the most frequently cited as least impactful category at both T2 (22.2%) and T3 (24.2%).

Among the open-ended questions, participants were asked what their understanding of postpartum doula care was. Responses frequently included the word “help” in the context of obtaining support and relief. Help was noted in terms of practical assistance with baby, household tasks and their own recovery. Notably, several participants recognized the potential for doula care to support their mental health.

At the end of the intervention (T3), future acceptability of postpartum doula care was also strongly endorsed. The majority (84.0%) of participants expressed willingness to seek postpartum doula support again with a future child, and most (88.0%) participants reported they were “very likely” if it were covered by insurance (See Table 3). Participants also reported navigating meaningful social and structural stressors that could influence context in which doula care is provided, including food insecurity, financial worry, social isolation, and a history of trauma.

In terms of feasibility, Sanctuary Doulas provided established infrastructure in terms of visit scheduling, team communication, and data collection systems, which allowed for seamless implementation of the intervention across participants and study staff. Some delay between initial eligibility screening and the first study visit was common, driven largely by communication barriers such as inconsistent phone or internet access, scheduling conflicts, and in some cases housing instability.

## Discussion

This pilot study suggests that in-home postpartum doula care was associated with meaningful reductions in symptoms of depression and anxiety and highly acceptable in our cohort of BIPOC and Latina birthing parents. Over the course of the intervention, depression and anxiety symptoms declined significantly, with large effect sizes observed. Participants also reported high levels of satisfaction with the intervention and strong endorsement of its positive influence on their postpartum experience, mood, and anxiety. Taken together, these findings suggest that postpartum doula care may represent a promising, equity-oriented strategy for addressing unmet perinatal mental health needs.

These findings are especially important in light of the well-documented continuity gap in postpartum care. In the U.S., postpartum care has historically been concentrated in clinical follow-up despite increasing recognition that recovery, infant feeding, sleep disruption, and emotional adjustments unfold over weeks to months in the home (27, 28). Professional organizations, maternal health advocates, and researchers have called for postpartum care to be reconceptualized as an ongoing process rather than a single visit (29), however, U.S. health systems often remain poorly structured to provide sustained home-based support during this period. Our findings suggest that postpartum doulas may help fill this continuity gap by extending care into the everyday environments where mental health challenges are experienced.

Our results also align with the growing body of literature and evidence regarding the impact of practical and emotional postpartum support on maternal well-being. While prior doula research has focused predominantly on birth doulas and clinical obstetric outcomes (18), this pilot intervention study shifts the attention to the postpartum period. The observed changes in mood and anxiety symptoms may respond to different potential mechanisms. First, postpartum doulas can assist with tasks and burdens including meals, laundry, light housekeeping, and newborn care, which can reduce daily stress (30). This type of instrumental support may be especially meaningful during periods of severe sleep disruption, which is a known contributor to depression and anxiety in the postpartum period (31). Second, doulas provide emotional validation (13), which can reduce isolation and help parents feel seen, supported, and capable. Third, informational support and newborn care education could improve self-efficacy and reduce uncertainty, especially for first-time parents. Lastly, resource navigation and referral support may help families engage with systems that otherwise may feel difficult to access. Although these mechanisms are theoretically plausible and supported by prior literature, our study was not designed to determine which specific aspects of postpartum doula care contributed to the observed improvements in depression and anxiety symptoms.

Our findings also demonstrate the evolving nature of postpartum needs. Household support was most frequently identified as the most impactful aspect of care at the mid-study point, and remained highly endorsed at the endpoint, while newborn care, feeding, and education became more prominent over time. This pattern may reflect a shift over time in postpartum priorities: in the early weeks, parents may be most overwhelmed by basic household demands, whereas as physical recovery progresses and daily routines become more established, infant care guidance and parental confidence-building become increasingly central concerns.

Across many cultural traditions, the postpartum period has been recognized as a time that requires structured support, rest, nourishment, and temporary redistribution of domestic labor. Ethno kinship-based traditions such as *la cuarentena* in Latin America, *zuo yuezi* in Chinese traditions and postpartum ayurvedic care models all emphasize the need for care during maternal recovery (32). Although these practices differ substantially in their origins, beliefs, and specific practices, they share a broader recognition that postpartum wellbeing depends not only on medical monitoring but also on sustained social, emotional and practical support. In contrast, many birthing parents in the U.S. navigate the postpartum period within individualized household structures with limited access to ongoing community-based support beyond intermittent medical visits. In this context, postpartum doulas may help address this gap by providing professional, non-medical social, emotional and practical support that can complement clinical care and promote maternal wellbeing.

The novelty of this study lies in its focus on postpartum doula care as an equity-oriented approach to addressing unmet needs. BIPOC and Latina birthing parents face a disproportionate burden of PMHDs while also encountering greater barriers to diagnosis, treatment, and respectful care. This type of home-based, non-medical intervention may offer a valuable complement to conventional services to populations who experience logistical barriers in access and medical mistrust. As Medicaid programs and state policymakers increasingly explore reimbursement for doula services, these findings offer preliminary support for the potential of postpartum support as a maternal mental health equity strategy.

Several limitations warrant consideration. First, the study used a single-arm longitudinal design without a control group, which limits causal inference. Improvements over time may also partially reflect natural symptom resolution, regression to the mean, or other concurrent changes unrelated to the intervention. This is particularly relevant given that mild to moderate postpartum depression and anxiety symptoms may improve over time in some individuals even without intervention. However, some research has found that trajectories of depression and anxiety symptoms increase postpartum (33), and that the prevalence of depression symptoms remains high at 12 months postpartum (34). Second, the modest sample size reduced statistical power and limited the possibility of conducting subgroup analyses. Third, the reliance on self-reported measures for anxiety and depression may introduce reporting biases. Fourth, attrition and incomplete follow-up reduced sample sizes across timepoints, and participants who remained in the study may have differed from those who did not complete all assessments. Additionally, the qualitative component of this study was limited to a brief descriptive review of open-ended responses and should not be interpreted as a formal content analysis. Finally, because the sample was drawn from one metropolitan region in Colorado and intentionally focused on BIPOC and Latina families experiencing symptoms of depression and/or anxiety, findings may not generalize to all postpartum populations or settings.

Despite these limitations, this study addresses an understudied intervention and centers populations that bear disproportionate barriers postpartum mental health wellbeing. By using a pragmatic intervention that is home-based and adaptable, it is potentially scalable within community-based maternal mental health programs if further evidence confirms effectiveness. Future research should build on these preliminary findings through adequately powered randomized controlled trials or other strong comparative designs to better understand the effectiveness of the intervention and generate evidence needed to inform future implementation and policy decisions. Further work should also examine potential mechanisms of impact, including whether improvements are mediated by increased rest, reduced domestic strain, enhanced parenting confidence, stronger perceived social support, or improved linkage to services. Additional implementation research would also be important to determine how postpartum doula care can be integrated into Medicaid, safety-net systems, and culturally-responsive community-based models without losing the relational quality that participants appear to value most.

## Conclusion

This pilot study provides preliminary evidence that postpartum doula care may reduce symptoms of depression and anxiety among BIPOC and Latina birthing parents. Participants reported high levels of satisfaction, strong engagement, and broad acceptability of this home-based, non-medical intervention, supporting the feasibility and acceptability of further evaluating postpartum doula care through larger implementation and effectiveness studies. By extending support beyond the medical setting and into the home, postpartum doula care may help address important gaps in continuity of postpartum care while responding to the practical, emotional, and structural challenges that shape maternal mental health. Although larger randomized control studies are needed, these findings suggest that postpartum doula care is a promising strategy that warrants evaluation for scalability and its potential role in advancing perinatal mental health equity.

## Data Availability

The datasets presented in this article are not readily available due to the small sample size and the sensitive nature of the data collected from postpartum BIPOC and Latina participants. The dataset is not publicly available and cannot be shared upon request because of the risk of deductive disclosure and potential loss of participant confidentiality. Requests to access the datasets should be directed to JH, hello@sanctuarycares.org.
